# GETCO2: A Compact and Portable System for Automated Gas Transfer Velocity Measurements of Natural Waters

**DOI:** 10.1021/acsearthspacechem.5c00329

**Published:** 2026-02-24

**Authors:** Sevda Norouzi, Ryan Pereira

**Affiliations:** The Lyell Centre, School of Energy, Geoscience, Infrastructure and Society, 3120 Heriot-Watt University, Research Avenue South, Edinburgh EH14 4AS, United Kingdom

**Keywords:** air−water CO_2_ flux, surfactant suppression effect (SSE), carbon cycle, automated gas exchange tank, greenhouse gas exchange

## Abstract

To enhance our understanding of surfactants in the exchange of climatically active gases across the water–atmosphere interface, we introduce a novel CO_2_ gas exchange tank equipped with a gas transfer velocity (*k*_w_) estimation tool, significantly improving upon earlier designs. This advanced platform integrates a commercially available, cost-effective, high-performance nondispersive infrared (NDIR) CO_2_ sensor with a closed controlled environment gas exchange tank. The tank is designed to induce precisely controlled water-side turbulence, allowing for the exploration of biological and chemical factors controlling the *k*_w_. The new system features a bespoke 3D-printed automated equilibrator, enabling precise measurements of the water CO_2_ concentration. CO_2_ concentrations in both air and water phases are measured using a novel setup that minimizes errors from sensor drift and incomplete mixing or equilibration. Measurement errors for equilibrated CO_2_ concentrations in air and water are 1.3 and 3.4%, respectively. The precision achieves *k*_w_ values of 7.21 ± 0.18 cm h^–1^ with a coefficient of variation (CV) of 2.5% for deionized water. Testing with freshwater and marine waters yielded *k*_w_ suppression levels comparable to an earlier automated gas exchange tank system utilizing gas chromatography. This next-generation GETCO2 system is smaller, easier to maintain, and more portable than previous models, allowing for wider-scale studies aimed at investigating the impact of surfactants and other organic compounds on *k*_w_ suppression and enhancement across various natural water environments.

## Graphical abstract

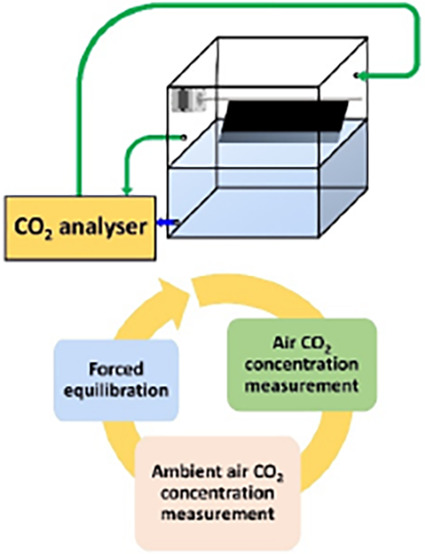

## 1 Introduction

Oceans are a global reservoir of greenhouse gases and are estimated to account for 20–40% of the postindustrial sink for anthropogenic carbon dioxide (CO_2_)^1^ and are estimated to store ∼3 petagrams (Pg) of carbon (C) per year.^2^ However, quantifying the exchange of key greenhouse gases such as CO_2_, methane (CH_4_), and nitrous oxide (N_2_O) across the air–water interface of the ocean is a major environmental, logistical, and methodological challenge. For CO_2_, the uncertainty of oceanic storage is estimated at ±0.6 Pg C yr^–1^ and is primarily derived from spatial and temporal uncertainty in the gas transfer velocity (*k*_w_). This variability complicates gas flux estimates and may reflect variable enrichments in surface-active organic matter (surfactants) that cause turbulence suppression in the uppermost layer of the ocean, known as the sea surface microlayer (SML)—the skin of the ocean.^3^ Establishing the changing rate of gas exchange through space and time and understanding the drivers are critical to estimate the intrinsic oceanic sinks and sources of these key greenhouse gases.

The oceanic uptake rate of greenhouse gases is determined by relative differences in gas concentrations of water and air and the *k*_w_, which are controlled by spatial and temporal variability of near-surface turbulence. Environmental control of the *k*_w_ is exerted through the modification of turbulent diffusion at the air–sea interface.^4^ Wind speed is a fundamental control of near-surface turbulence but known to be a weak *k*_w_ predictor,^5^ with more inclusive *k*_w_ parametrizations compromised by insufficient descriptions of key controlling variables.^4^ The result is several parametrizations^6–10^ with differences that imply variable modifications from other factors such as atmospheric stability, sea state, breaking waves, white caps, bubble transport, rain, and the presence of organics in the SML.^11^ The SML (depth <400 μm) is a physically and biogeochemically distinct ocean–atmosphere interface covering the entire ocean surface.^12^ SML enrichments in the dissolved material and buoyant particles are ubiquitous, with the surfactant concentration often greater in oligotrophic than in more productive areas and persisting to wind speeds of at least 13 m s^–1^ ^13,14^ with no significant depletion beyond 5 m s^–1^.^14^ SML enrichment occurs mainly via bubble scavenging from underlying water,^3,11,12,14,15^ leading to increased microbiological and photochemical processing.^3,16^

Reduced gas exchange by the suppression of the *k*_w_ from surfactants, known as the “surfactant suppression effect” (SSE), has been shown to reduce the amount of CO_2_ annually stored by ∼9% in the Atlantic Ocean^17^ and to control the spatiotemporal flux of N_2_O.^18^ Importantly, *k*_w_ suppression has a large spatial variability that could mean CO_2_ storage in key regions have a high uncertainty where purely wind-based *k*_w_ parametrizations are used.^17,19^ A decrease in the *k*_w_ in the open ocean compared to coastal waters likely reflects a higher surfactant concentration (associated with higher productivity) nearshore^20^ and/or a large source of terrestrial DOM.^14,19,21^ However, clear linkages between Chlorophyll a (commonly used as a proxy for primary productivity) and the total surfactant are not always apparent.^13,17,22^ Furthermore, universal relationships between the *k*_w_ and the total surfactant, or between the *k*_w_ and either the wind speed or the capillary wave slope for surfactant-covered waters, have proven elusive. Work in the California Bight found strong relationships between the SA, the wind speed, and the capillary wave slope,^23^ but laboratory studies found no link between surfactants and capillary waves.^24^ This lack of a universal relationship must reflect strong spatiotemporal gradients fueled by varying surfactant sources (e.g., terrestrially derived organic matter vs marine in situ productivity) that are likely associated with seasonality.

Despite the growing recognition of the surfactant suppression effect, quantifying its impact on air–water gas exchange remains challenging due to the spatial and temporal variability in surfactants and the difficulty of isolating these effects in situ. Gas exchange tanks offer a controlled environment to investigate *k*_w_ suppression under isolation. The automated tank developed by ref 25 provided a robust platform for measuring the *k*_w_ using multiple tracer gases, enabling a direct comparison between natural seawater and surfactant-free controls.^17,19^ However, this system was challenging to operate, requiring full-scale laboratory conditions including a large footprint, significant power supply, and large gas supplies. To build on the foundation of this system and overcome these barriers for broader-scale implementation, we present a new portable gas exchange tank and CO_2_ sensor system (GETCO2) designed to estimate the gas transfer velocity of CO_2_ with enhanced utility and operational flexibility. The GETCO2 system integrates a next-generation nondispersive infrared (NDIR)-based analyzer to measure CO_2_ with real-time data acquisition, enabling high-resolution measurements of the *k*_w_ across a range of natural water conditions. Importantly, it allows for deployment in coastal and remote environments where surfactant variability is pronounced and where traditional laboratory infrastructure (space and power) is limited. This paper describes the design, calibration, and validation of the GETCO2 system and demonstrates its utility in resolving fine-scale variability in the *k*_w_ linked to surfactant activity. By enabling portable, high-precision measurements of the CO_2_ exchange, GETCO2 contributes to the development of more accurate biogeochemical models and supports efforts to refine regional and global assessments of ocean–atmosphere carbon flux.

## 2 Methodology

### 2.1 System Design

The GETCO2 system is specifically designed to ascertain the relative impact of SML modulation through surfactants. As such, the tank must be small enough to ensure the adequate cleaning protocol between samples while being large enough to ensure microlayer formation and gas tracer analysis. While the absolute *k*_w_ values may not be comparable to in situ conditions, comparative *k*_w_ measurements at constant turbulence, normalized to surfactant-free water, permit the adaptation of commonly employed wind-based parametrizations to factor the impact by surfactants.^17^ In combination with field-based measurements such as eddy covariance and dual tracer methods^26,27^ with controlled laboratory gas exchange tanks and in situ floating platform measurements,^17,28^ a holistic and mechanistic understanding may be achieved, enabling more precise parametrizations of gas exchange.

Figure 1a shows the schematic of GETCO2, which includes a water leveler assembly, a gas exchange tank, a baffle assembly, and a CO_2_ analyzer. The CO_2_ analyzer includes the fluid pathways, an NDIR CO_2_ analyzer, a valve and pump control unit, a power supply, and an automated equilibration system. Air, water, and brine are pumped into the flow lines, and solenoid valves control the pathways of the fluid (see below for more details). All of the fluid pathways in the water leveller assembly and the tubes from the tank to the CO_2_ analyzer are sealed and made from stainless steel (SS) or plastic components like HDPE, vinyl, polypropylene, and poly­(vinylidene fluoride) (PVDF) to minimize corrosion or contamination potential. A key benefit of the GETCO2 system is the automation of gas measurements in the water and air phases of the tank to calculate the *k*_w_. The GETCO2 measurement cycle (Figure 1b) begins by expelling water and gas from the measurement circuits. The sensor shell is rinsed with the air from the tank headspace, and the CO_2_ concentration is continuously measured until the variance is less than the sensor sensitivity (typically 4 ppmv in less than 40 s). Once complete, the CO_2_ concentration of the ambient air outside of the tank is measured to ascertain a correction factor for the equilibrator measurement. Concomitantly, the equilibrator is filled with sample water from the tank for degassing with ambient air and sparged until a CO_2_ concentration is established that has less variance than the sensor sensitivity (typically 3 min).

**Figure 1:**
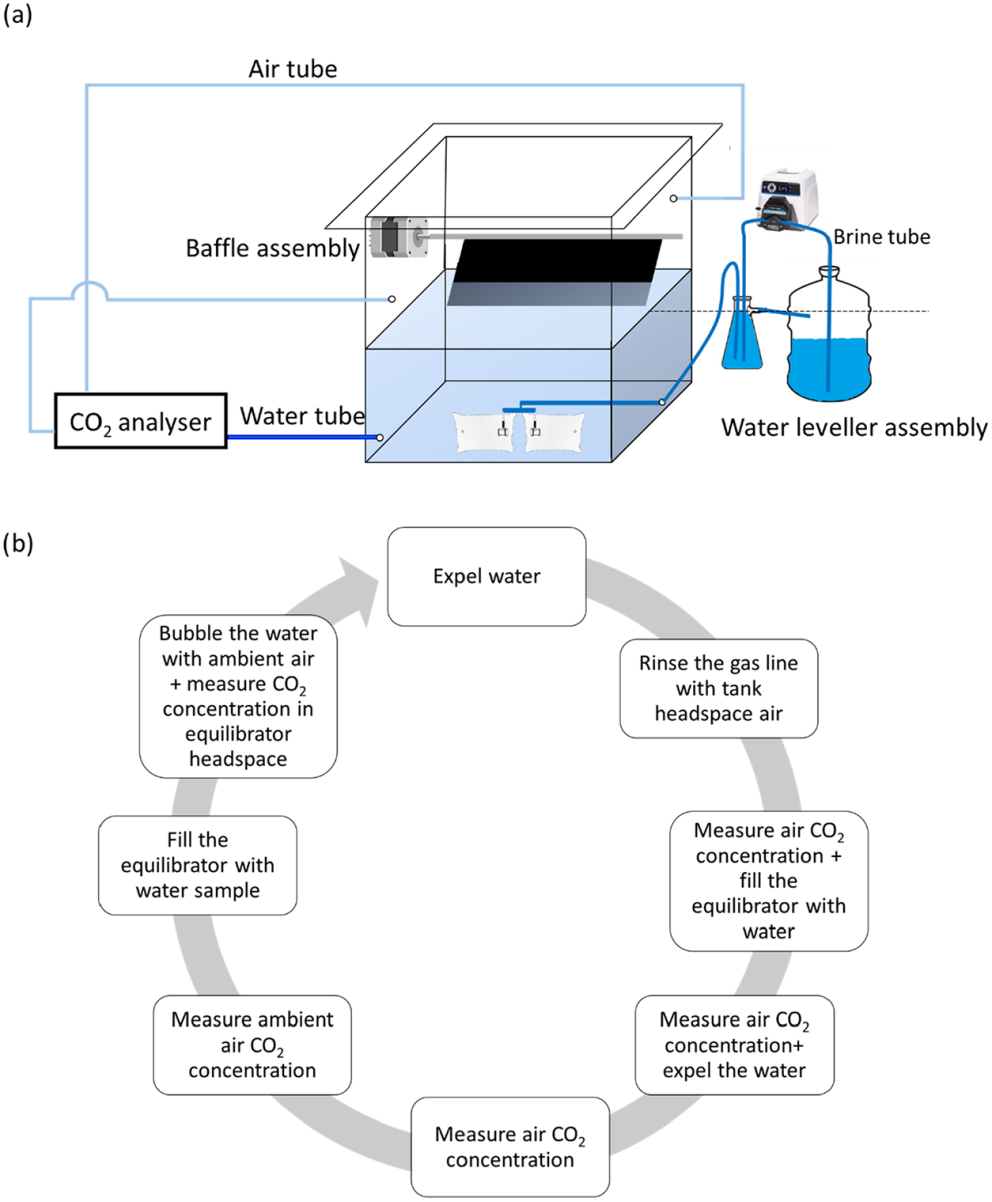
(a) Schematic of GETCO2 including the water leveller assembly, gas exchange tank, baffle assembly, and CO_2_ analyzer. Brine, water, and air pathways (tubes) are marked with different shades of blue. (b) CO_2_ analyzer measurement cycle.

### 2.2 Gas Exchange Tank

The gas exchange tank of the GETCO2 system (Figure 1a) is based on the original design presented by ref 29 and constructed using 12 mm-thick clear Perspex acrylic sheets with inner dimensions of 42, 42, and 45 cm (W, L, and H). The tank lid is sealed by 40 stainless steel (SS) screws and wingnuts and a gasket located between the lid and its seating plate to ensure that the tank is gastight. The gas/water in/outlets of the tank are sealed using SS bulkhead fittings. The tank is filled with water samples to a volume of 38.8 L leaving a 40.5 L headspace. A headspace pressure relief valve is placed on the lid to stop overpressurization and subsequent damage to the tank structure in the event of system malfunction. This water leveller assembly replaces the water volumes and is removed from the tank by the CO_2_ analyzer to measure the CO_2_ concentration in water, with brine (NaCl (aq)) inside expandable plastic bags to keep the water level and pressure inside the tank constant during the experiments following the previously introduced protocols.^25,29^ The density of the brine inside the expandable plastic bag is always kept higher than the water sample to ensure a negative buoyancy to prevent it from rising to the water surface and impacting the SML.

### 2.3 Baffle Assembly

A baffle assembly is used to create reproducible turbulence in the tank to facilitate gas exchange between water and air. The baffle assembly includes an Arduino board that controls a bipolar stepper motor driver outside the tank. A single SS cylinder shaft connects the motor to the water SS baffle. The motor (PD60–3–1276-CANOPEN) operates in a bidirectional, stepwise manner, moving the baffle between predefined angular positions before reversing the direction, resulting in a periodic oscillatory motion. The baffle motion frequency is fixed (0.20 ± 0.02 Hz) for each experiment and controlled programmatically via the stepper motor settings, as described by Schneider-Zapp et al.^25^ The objective of this design is not to replicate the full complexity of wind-wave dynamics but to provide a controlled and repeatable turbulence field suitable for comparative gas exchange measurements. Bearings were used at both ends of the shaft for a gastight seal. The motor can be powered by either a 24 NiMH rechargeable battery size C 1.2 V with the capacity of 4500 mAh connected in series or a 24 V 3A DC power supply.

### 2.4 Selection of CO_2_ as a Trace Gas

In previous generations of the gas exchange tanks, SF_6_, CH_4_, and N_2_O were commonly used as trace gases.^25^ Here, we selected the direct analysis of CO_2_, which has two main advantages. First, the direct measurement of CO_2_ removes the potential issue of Schmidt number scaling inaccuracies from using tracer gases (based on either fugacity or surface turbulence), as gases of interest are directly determined.^30,31^ Second, the measurement times using NDIR are <10 s compared to 6–8 min using gas chromatography, allowing for near-simultaneous measurement of gases in the atmosphere and water (via an equilibrator step). This ensures that the water sample is fully equilibrated during analysis through real-time analysis and allows the same NDIR sensor to be used for both air and water phases, further reducing analytical uncertainties.

### 2.5 Equilibrator

The NDIR sensor used in GETCO2 is suitable to measure only CO_2_ in the gas phase. To utilize the same sensor for air and water, GETCO2 automatically samples the water phase at discrete time intervals. This aliquot of water degassed in a sealed equilibrator with ambient air provided a gas sample suitable for CO_2_ measurement using the NDIR sensor. The process of using forced equilibration for the measurement of concentration in water has been shown to be highly reproducible for SF_6_, N_2_O, and CH_4_ gases.^32^ Due to the challenges of creating a gastight and robust equilibrator constructed from glass for field deployment, an equilibrator was designed and 3D-printed using ABS plastic. The equilibrator was sealed with sealant paste and tested for gas leakage under a 3 atm pressure using certified CO_2_ standard gases.

The performance of the GETCO2 equilibrator was assessed using 18.2 MOhm, surfactant-free deionized water, two standard gases (10 and 1000 ppmv of CO_2_), and the ambient lab air (400 ppmv of CO_2_). For each gas, water was sparged through three equilibration cycles, with the measured values reported in Table 1. Across all nine measurements, the average water CO_2_ concentration was 565 ppmv (SD = 19 ppmv; CV = 3.4%). These overall results demonstrate the high precision of the forced equilibration method, independent of the gas used. The relative uncertainty for water CO_2_ measurements is 3.4%.

**Table 1:** Equilbrator Performance Test Results of Deionized Water

certified gas (ppmv)	cycle 1 (ppmv)	cycle 2 (ppmv)	cycle 3 (ppmv)	mean (ppmv)	SD (ppmv)
10	579	579	568	575	6
1000	579	558	521	552	29
ambient (400)	580	555	566	567	12

### 2.6 CO_2_ Analyzer

A K30 NDIR CO_2_ sensor is used to measure CO_2_ in air and equilibrator headspace (CO2Meter, UK), which has been successfully deployed in field-based analyzers.^33–35^ The sensor measurement is based on diffusion with a linear range from 0 to 10,000 ppmv CO_2_ with a response time of 2 s for a 0.5 L min^–1^ gas flow. To control the sensor, valves, and pumps of the system, an Arduino board was selected as an open-source electronic platform. The Arduino board processes and stores all sensor inputs and controls the gas and water flow pathways. The microcontroller operates valves and pumps by a common insulated gate field effect transistor (MOSFET) with the voltage supplied as specific to the valve or pump. An SD card module is used to write the sensor measurements to a flash memory card or can be read directly from Arduino interface software via a connected laptop. The system stores the time and measurements from the sensor and the final values of the measured air CO_2_ concentration and equilibrated headspace CO_2_ concentration in separate.txt files. The Arduino board of the CO_2_ analyzer is powered using either a 9 V supply or a standard laptop computer. All additional circuits are powered by rechargeable batteries or mains power, depending on the application setting (i.e., lab vs field use).

### 2.7 CO_2_ Sensor Accuracy and Precision

Calibration of GETCO2 is conducted without water in the system. The CO_2_ sensor is calibrated using three certified standard gases at 10, 1000, and 3000 ppmv, respectively (BOC, UK), to generate a linear calibration (Figure 2). As each flow path is pumped separately to the sensor shell, three separate calibrations for air, ambient air, and equilibrated water are determined to minimize errors. Figure 2 shows a typical calibration with a *R* ^2^ greater than 0.99 for each flow path. The limit of detection for CO_2_ in GETCO2 corresponds to the lowest concentration of the CO_2_ gas used in the calibration of 10 ppmv.

**Figure 2:**
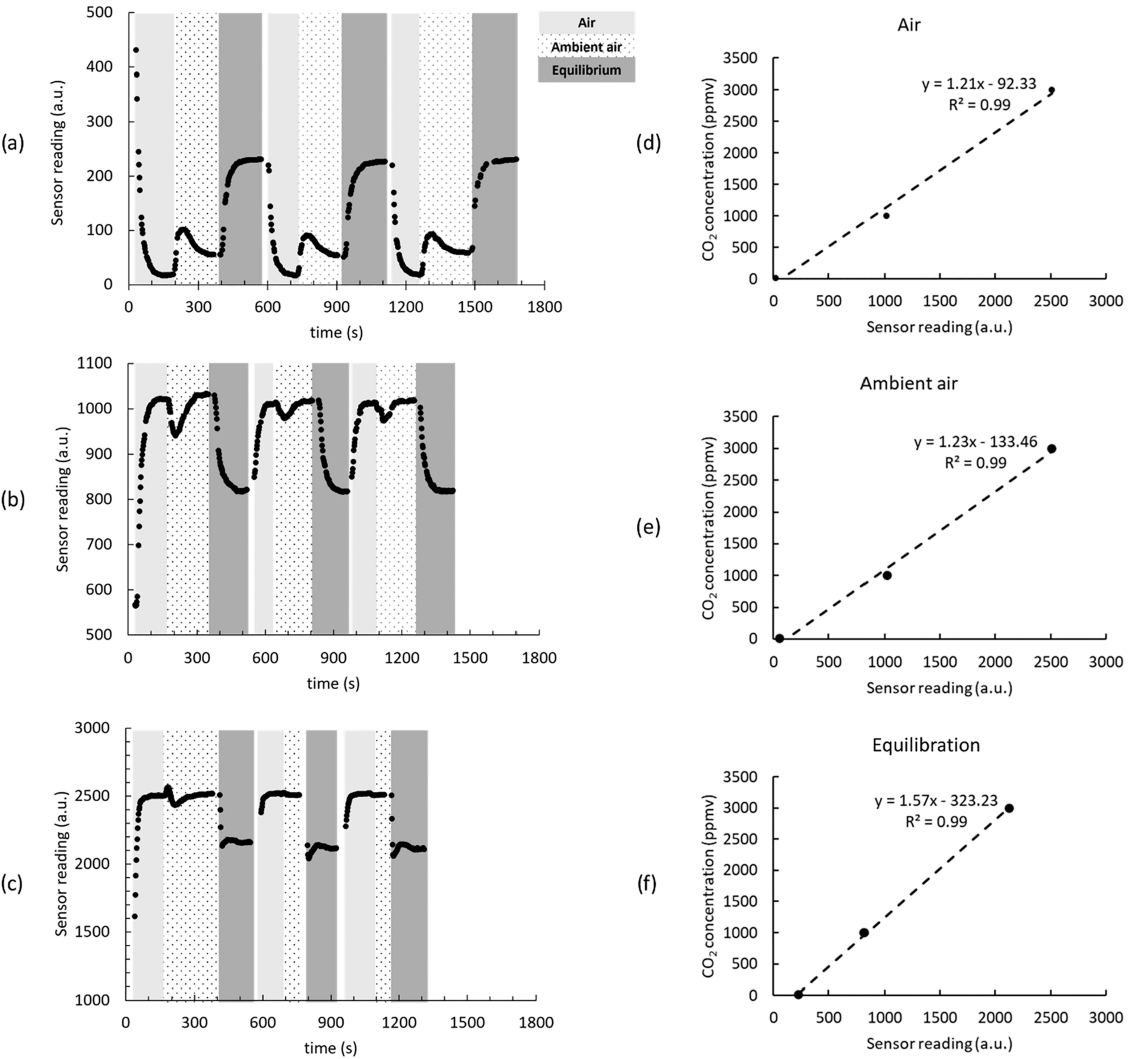
CO_2_ sensor readings for (a) 10, (b) 1000, and (c) 3000 ppmv CO_2_ standard gas and CO_2_ calibration results for (d) air, (e) ambient air, and (f) equilibration steps of the analytical cycle.

To define the precision of the GETCO2 system, the 1000 ppmv certified CO_2_ reference gas was used. The equilibrium step (clean equilibrator, without water) was run five times with the standard gas for at least 30 min. The final 20 measurements of each run represent the final measured value and the range between maximum and minimum values defined as the sensor sensitivity. This procedure was repeated for the gas tank air phase and ambient air CO_2_ measurement steps to define the individual sensitivities of each flow path separately. The number of the measurements for the sensitivity criteria can be changed based on the concentration range and variation. The reported error for the air, ambient air, and equilibrator measurements are 5.2, 4.2, and 2.8%, respectively.

## 3 Experimental Protocols

### 3.1 Preparation

Prior to sample analysis, the tank and tubing are first prerinsed with deionized water, cleaned with 99% ethanol, and flushed further with deionized water. Potential sample contamination and carryover effects are minimized by analyzing the rinsate for total surfactant activity (SA)^19,36^ until the measurement corresponds to the blank deionized water. Experiments were run by saturating a 1.9 L aliquot of sample water with 100 mL of 99.9% CO_2_ for 30 min with the resulting solution added to the tank.^25^ The water height was set at 22 cm for all experiments, and the tank was sealed under ambient pressure. The water was left for 15 min to become well mixed before beginning the experiment. The volume of the water removed via the equilibrator during the experiments was recorded to calculate the total mass balance of CO_2_ throughout the experiment.

### 3.2 *k*_w_ Calculation

Based on the CO_2_ concentration in the air and equilibrated headspace air, the water CO_2_ concentration can be calculated using the following equation

1 $$C_{w}=\frac{\left(\left(V_{h}+{\alpha V}_{\mathrm{ws}}\right)\times C_{e}-{V_{h}C}_{\mathrm{aa}}\right)}{V_{\mathrm{ws}}}$$

where *V*_h_ is the headspace volume, *V*_ws_ is the equilibrator water sample volume, *C*_e_ is the CO_2_ concentration in the equilibrated headspace air, and *C*_aa_ is the CO_2_ concentration in ambient air.

The flux of slightly soluble gases such as CO_2_ across the water–atmosphere interface can be defined as eq 2

2 $$F=k_{w}\left({\alpha C_{a}-C}_{w}\right)$$

where *F* is the flux (mol m^–2^ s^–1^), *k*_w_ is the gas transfer velocity (m s^–1^), α is the Ostwald solubility coefficient, and *C*_a_ is the gas concentration in air (mol m^–3^).^37^ α can be estimated using the following equation

3 $$\alpha =K_{0}RT_{w}$$

where *K*_0_ (mol m^–3^ Pa^–1^) is the aqueous-phase solubility of CO_2_, *R* (m^3^ Pa K^–1^ mol^–1^) is the ideal gas constant, and *T*_w_ (K) is the water temperature.^37^ *K*_0_ is calculated by eq 4

4 $$\ln K_{0}=A_{1}+A_{2}\left(\frac{100}{T_{w}}\right)+A_{3}\ln\left(\frac{T_{w}}{100}\right)+S\times \left(B_{1}+B_{2}\left(\frac{T_{w}}{100}\right)+B_{3}{\left(\frac{T_{w}}{100}\right)}^{2}\right)$$

where *K*_0_ is in mol L^–1^ atm^–1^, *A*’s and *B*’s are constants (Table 2), and *S* is the salinity in ppt.^38^

**Table 2:** Constants of Aqueous-Phase Solubility (*K*_0_) versus Temperature Equation

*A*_1_	*A*_2_	*A*_3_	*B*_1_	*B*_2_	*B*_3_
–58.0931	90.5069	22.2940	0.027766	–0.025888	0.0050578

As GETCO2 is a closed tank, the mass balance equations will be as follows

5 (dCadtVadCwdtVw)=kw(Cw−αCaαCa−Cw)A

where *V*_w_ is the water volume (m^3^),*V*_a_ is the air volume (m^3^), and *A* is the surface area (m^2^). Assuming $h_{i}=\frac{V_{i}}{A}$ and *D* = *C*_w_ – α*C*_a_, eq 5 will result in

6 $$\frac{dC_{a}}{dt}=\frac{1}{\alpha }[\frac{dC_{w}}{dt}-\frac{dD}{dt}]=\frac{k_{w}}{h_{a}}D$$

Using the bottom equation of eq 5 in eq 6, a differential equation in *D* is obtained as follows

7 $$\frac{dD}{dt}+k_{w}\left[\frac{1}{h_{w}}+\frac{\alpha }{h_{a}}\right]=0$$

Assuming $\beta =\frac{1}{h_{w}}+\frac{\alpha }{h_{a}}$ , the differential equation is

8 $$\frac{dD}{dt}+k_{w}\beta D=0$$

The solution for eq 8 is as follows

9 $$D=D_{0}\exp\left(-k_{w}\beta t\right)$$

where *D*_0_ = *D*(*t* = 0). Therefore

10 $$\frac{\ln\left(\frac{D}{D_{0}}\right)}{\beta }=k_{w}t$$

Assuming $L=\ln\left(\frac{D}{D_{0}}\right)$

11 $$\frac{L}{\beta }=k_{w}t$$

The *k*_w_ can be estimated by a linear fit to eq 11. Gaussian error propagation for $\frac{L}{\beta }$ is presented in Appendix A (Supporting Information for publication).

GETCO2 is a closed tank system, and in each sampling time (*t_n_*), the actual volume of water will change. The volume of the water sample or air in the tank is

12 $$V_{ms_{n}}=V_{mt_{n-1}}-V_{mt_{n}}$$

where *m* refers to water or air, and *V_ms_n__*__+1__ is the volume of the fluid *m* removed from the tank for sample *n* + 1 at *t*_*n*+1_. Assuming a constant surface area

13 $$h_{ms_{n}}=h_{mt_{n}-1}-h_{mt_{n}}$$

14 $$h_{mt_{n}}=h_{mt_{n-1}}-h_{ms_{n}}$$

15 $$h_{mt_{n}}=h_{m0}-\sum_{i=1}^{n}h_{\mathrm{mi}}$$

16 $$\beta_{j}=\frac{1}{h_{w0}-\sum_{i=1}^{j}h_{ws_{i}}}+\frac{\alpha }{h_{a0}-\sum_{i=1}^{j}h_{as_{i}}}$$

The only step where GETCO2 removes air from the tank is during rinsing of the sensor shell, when fresh air from the tank’s headspace is pumped in to replace the air from the previous steps. This process uses the same volume of air for all cycles

17 $$V_{\mathrm{as}}={\mathrm{Ah}}_{\mathrm{as}}$$

18 $$h_{\mathrm{as}}=V_{\mathrm{as}}/A$$

This changes β; thus, eq 8 has no constant coefficients.

The removed water volume can be calculated by the product of the rate of the water pump and its duration.

19 $$h_{ws_{n}}=\frac{t_{pn}Q_{p}}{A}$$

20 $$\beta_{j}=\frac{1}{h_{w0}-\frac{Q_{p}\sum_{i=1}^{j}t_{pi}}{A}}+\frac{\alpha }{h_{a0}-jh_{\mathrm{as}}}$$

where *t*_p*n*_ is the duration of the water pump working (s) at cycle *n* and *Q*_p_ is the pump rate (m^3^ s^–1^). The volume of the wastewater is measured to calculate *Q*_p_ using eq 19

21 $$Q_{p}=\frac{V_{\mathrm{wE}}+V_{\mathrm{wL}}}{t_{p}}$$

where *V*_wE_ is the wastewater volume expelled from the equilibrator (bottom outlet), *V*_wL_ is the wastewater volume from the level valve of the equilibrator (middle outlet), and *t*_p_ is the total time the pump worked while collecting the wastewater.

As the water leveler assembly keeps the water height constant, the air height is constant. Hence

22 $$\beta_{j}=\frac{1}{h_{w0}-\frac{Q_{p}\sum_{i=1}^{j}t_{pi}}{A}}+\frac{\alpha }{h_{a0}}$$

To solve the differential equation, it follows that

23 $$D_{n}=D_{n-1}\exp\left(-k_{w}\beta_{n}\left(t_{n}-t_{n-1}\right)\right)$$

24 $$D_{n}=D_{0}\;\exp\left(-k_{w}\left[\sum_{j=1}^{n}\beta_{j}\frac{\left(t_{j}-t_{j-1}\right)}{\left(t_{n}-t_{0}\right)}\right]\left(t_{n}-t_{0}\right)\right)$$

Assuming

25 $$B_{n}=\sum_{j=1}^{n}\beta_{j}\frac{\left(t_{j}-t_{j-1}\right)}{\left(t_{n}-t_{0}\right)}$$

The *k*_w_ can be determined by a linear fit to the following equation

26 $$\frac{L_{n}}{B_{n}}=k_{w}\left(t_{n}-t_{0}\right)$$

where $L_{n}=\ln\left(\frac{D_{n}}{D_{0}}\right)$

Gaussian error propagation for $\frac{L_{n}}{B_{n}}$ is presented in Appendix B (Supporting Information for publication).

## 4 Results and Discussion

### 4.1 GETCO2 Baseline Performance

To establish a baseline of the GETCO2 system and assess the precision of *k*_w_ measurements, deionized water was tested in triplicate under identical baffle settings. Figure 3a shows the CO_2_ concentrations in water and air during an experiment with supersaturated deionized water. Over 65 min, the water CO_2_ concentration decreased from 4245 to 2961 ppmv, while the air CO_2_ concentration increased from 770 to 2121 ppmv. Figure 3b presents the total CO_2_ in the tank normalized to the initial measurement. The mass balance remained within 3% throughout the experiment, consistent with previous studies.^17,19^ These small deviations fall within the measurement error, confirming that the system was gastight with no loss or gain of water or gas. Such mass balance checks are critical, as any leakage could lead to an overestimation of the *k*_w_. From the slope of $\frac{L_{n}}{B_{n}}$ vs time (Figure 3c), the measured *k*_w_ was 6.79 cm h^–1^. At a water temperature of 15.9 °C, this corresponds to a *k*_660_ value of 7.17 cm h^–1^. Two replicate experiments yielded *k*_660_ values of 7.41 and 7.06 cm h^–1^. Thus, the average *k*_660blank_ for deionized water (22 cm depth) under the specified motor settings (300 steps/s speed, 200 steps/s^2^ acceleration, and 200-step bounce) is 7.21  ±  0.18 cm h^–1^ (RSD = 2.5%). This precision is comparable to earlier gas exchange systems, which reported ∼4% accuracy for deionized water.^25^

**Figure 3:**
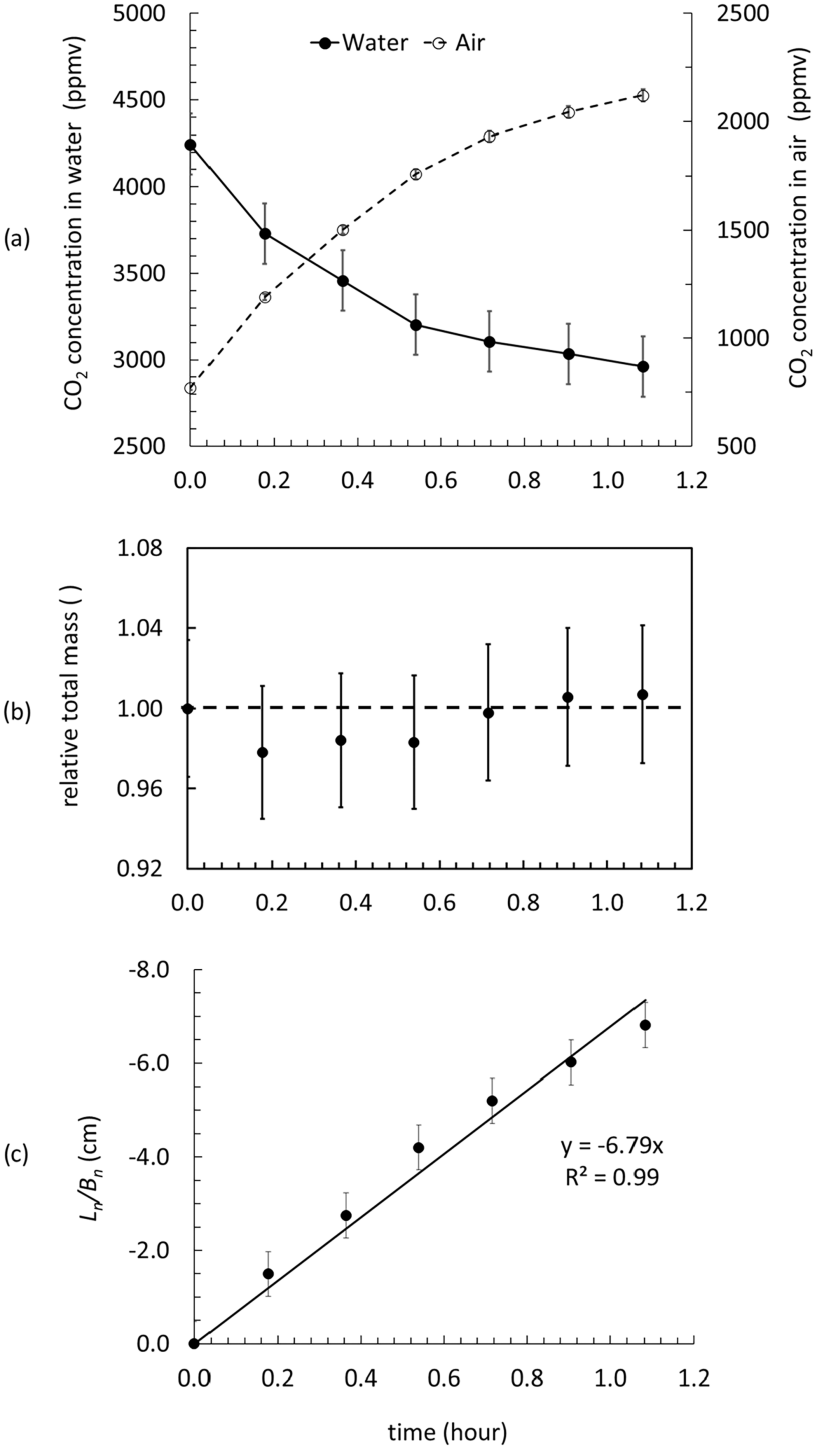
(a) CO_2_ concentration measured in water and air, (b) total amount of CO_2_ in the tank normalized to the first measurement, and (c) $\frac{L_{n}}{B_{n}}$ vs time for a typical GETCO2 experiment with deionized water. The error bars for the CO_2_ concentration in air and water are 1.3 and 3.4%, respectively. The dashed line in panel (b) shows that the relative total mass = 1. The error bar is 3.4% in panel (b). The water temperature was 15.9 °C for this experiment. The slope of the linear fit to panel (c) is estimated as *k*_w_ (cm h^–1^) (*k*_660blank_ = 7.17 cm h^–1^). The error bar is 0.48 cm in panel (c).

### 4.2 Field Experiments

To test GETCO2 in the field, marine waters and freshwater were investigated. The marine waters were collected from three locations in the North Atlantic Ocean (NAO) in 2022 from the *RRS Discovery* to compare with measurements in 2014.^17^ Samples were analyzed following the method outlined in ref 17. Briefly, a surfactant-free deionized water sample (blank sample) was run when the ship was stationary (referred to herein as an installation blank). Before each seawater sample experiment, another surfactant-free deionized water sample (sample blank) was analyzed to account for the general movement of the ship (*k*_660Sample_^′^ = *k*_660Sample_ × (*k*_660Installationblank_/*k*_660Sampleblank_)). To assess the SA effect, *k*_660Sample_^′^ was normalized to the *k*_660_ value of the installation blank (*R*_660_^′^ = *k*_660Sample_^′^/*k*_660Installationblank_).^17^ Freshwater samples were collected from a small lake and river (creek) located in the campus of Heriot-Watt University (HWU), Edinburgh, UK. These experiments were conducted at the Lyell Centre Wolfson Aquarium. Table 3 includes the details of locations, date, time, and water salinity and temperature for each sample. Aliquots of samples were collected for SA analysis following the method described in ref 36.

**Table 3:** Locations and Water Properties of the Water Samples Used to Test GETCO2

station name	longitude	latitude	date	salinity (PSU)	water temperature (°C)	SA (mg L^–1^ T-X-100)
NAO_1	24.93673	–21.27052	21/02/2022	36.99	21.29	0.31
NAO_2	27.62398	–14.19543	24/02/2022	36.67	18.86	0.28
NAO_3	27.62399	–14.19545	24/02/2022	36.67	18.86	0.09
HWU_Lake	55.9115	–3.3199	19/07/2023	0	15.5	1.81
HWU_Creek	55.9129	–3.3173	19/07/2023	0	14.1	1.68

### 4.3 Field Application and Testing of *R* Estimation

The SA effect on *k*_660Sample_ was normalized to *k*_660blank_ (*R* = *k*_660Sample_ */ k*_660blank_).^17,19^

The *k*_660blank_ experiment results from the North Atlantic Ocean ranged from 2.61 to 4.20 cm h^–1^. Once normalized for ship movement, the estimated *k*_660_ values for marine waters NAO_1, NAO_2, and NAO_3 were 0.58 ± 0.03, 0.67 ± 0.04, and 1.88 ± 0.06 cm h^–1^, respectively. The *R* values were 0.79 ± 0.07, 0.71 ± 0.05, and 0.74 ± 0.04, respectively. Therefore, 21, 29, and 26% suppression of *k*_660_ was observed for NAO_1, NAO_2, and NAO_3, respectively. The SML SA concentrations were 0.31 ± 0.07, 0.28 ± 0.09, and 0.09 ± 0.03 mg L^–1^ T-X-100 eq., respectively (Figure 4). *k*_660_ was always lower in seawater samples in comparison with blank waters corresponding to previous measurements from the region.^17^ The results from the lake and river showed that *k*_660_ = 4.73 ± 0.45 and 3.97 ± 0.28 cm h^–1^, respectively. The *R* values for these samples are 0.55 ± 0.06 and 0.66 ± 0.06, which indicate 34 and 45% suppression of the *k*_660_ of our test waters when compared to surfactant-free waters. The corresponding SML SA concentrations were 1.81 ± 0.02 and 1.68 ± 0.02 mg L^–1^ T-X-100 eq., respectively (Figure 4). Further work is required to better ascertain the implications of these measurements in the wider spatial and temporal contexts of freshwater systems.

**Figure 4:**
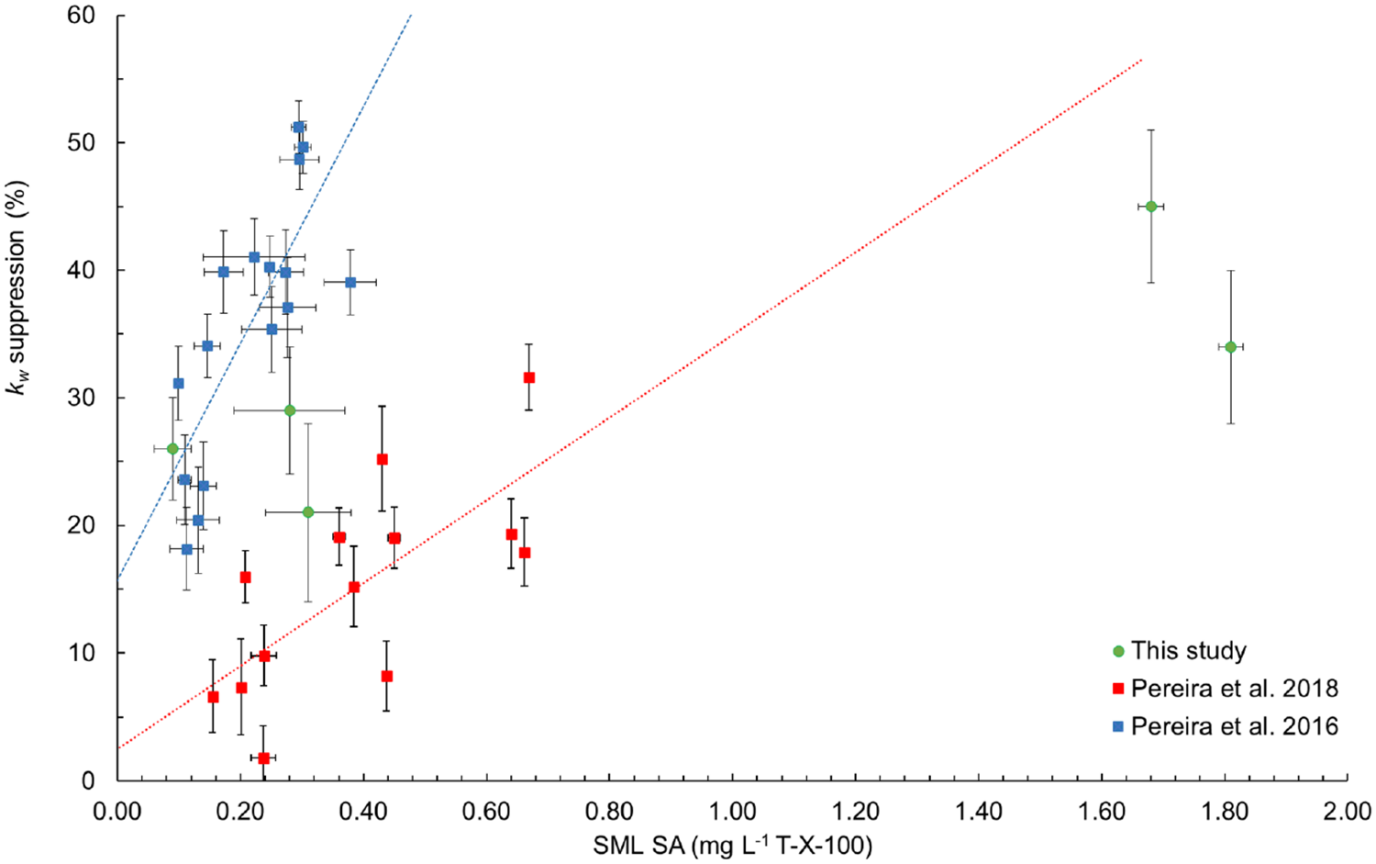
*k*_w_ suppression vs SA using data from refs 19 (blue squares),^17^ (red squares), and field samples from North Atlantic Ocean and freshwater samples (HWU_Lake and HWU_Creek) (green circles).

## 5 Conclusion and Implications

The GETCO2 system represents a significant advancement in the measurement of gas transfer velocities across the water–atmosphere interface. By integrating a cost-effective, high-performance NDIR CO_2_ sensor with a sealed, turbulence-controlled gas exchange tank, this platform enables the precise and reproducible estimation of the *k*_w_ in both marine and freshwater environments. The inclusion of a bespoke 3D-printed automated equilibrator and a novel dual-phase CO_2_ measurement setup minimizes common sources of error, achieving measurement precisions of 1.3 and 3.4% for air and water CO_2_ concentrations, respectively. With a coefficient of variation of 2.5% for *k*_660_ in surfactant-free deionized water and performance comparable to previous systems using gas chromatography, GETCO2 demonstrates both reliability and accuracy. Its compact design, ease of maintenance, and portability make it well suited for widespread deployment in diverse aquatic environments. This enables broader investigations into the role of surfactants and organic compounds in modulating gas exchange processes, which is critical for improving our understanding of climate-relevant gas fluxes. By facilitating high-resolution, field-ready measurements of *k*_w_ suppression and enhancement, GETCO2 provides a robust empirical foundation for linking point-scale observations with satellite-based Earth system models. This integration has the potential to refine global CO_2_ budgets and enhance our understanding of the complex biogeochemical dynamics at the air–water boundary.

## Supplementary Material



## Data Availability

The data utilized in this research study are available as Supporting Information documents within this manuscript.

## Notes

Gaussian error propagation formula and data sets used for the generation of Figures 2 and 3 (PDF)

## References

[1] Ciais, P. ; Sabine, C. ; Bala, G. ; Bopp, L. ; Brovkin, V. ; Canadell, J. ; Chhabra, A. ; DeFries, R. ; Galloway, J. ; Heimann, M. Contribution of Working Group I to the Fifth Assessment Report of the Intergovernmental Panel on Climate Change. In Climate Change : The Physical Science Basis; Carbon and Other Biogeochemical Cycles; Cambridge University Press: Cambridge, 2013.

[2] Woolf D., Shutler J., Goddijn-Murphy L., Watson A., Chapron B., Nightingale P., Donlon C., Piskozub J., Yelland M., Ashton I. (2019). Key uncertainties in the recent air-sea flux of CO2. Global Biogeochem. Cycles.

[3] Engel A., Bange H. W., Cunliffe M., Burrows S. M., Friedrichs G., Galgani L., Herrmann H., Hertkorn N., Johnson M., Liss P. S. (2017). The
ocean’s vital skin: Toward an integrated
understanding of the sea surface microlayer. Front. Mar. Sci..

[4] Upstill-Goddard R. C. (2006). Air–sea
gas exchange in the coastal zone. Estuarine,
Coastal Shelf Sci..

[5] Asher W. E. (2009). The effects
of experimental uncertainty in parameterizing air-sea gas exchange
using tracer experiment data. Atmos. Chem. Phys..

[6] Nightingale P. D., Malin G., Law C. S., Watson A. J., Liss P. S., Liddicoat M. I., Boutin J., Upstill-Goddard R. C. (2000). In situ
evaluation of air-sea gas exchange parameterizations using novel conservative
and volatile tracers. Global Biogeochem. Cycles.

[7] Wanninkhof R. (1992). Relationship
between wind speed and gas exchange over the ocean. J. Geophys. Res.:Oceans.

[8] Wanninkhof R., Knox M. (1996). Chemical enhancement
of CO2 exchange in natural waters. Limnol. Oceanogr..

[9] Wanninkhof R., Hitchcock G., Wiseman W. J., Vargo G., Ortner P. B., Asher W., Ho D. T., Schlosser P., Dickson M. L., Masserini R. (1997). Gas exchange, dispersion,
and biological productivity on the west Florida shelf: Results from
a Lagrangian tracer study. Geophys. Res. Lett..

[10] Wanninkhof R., McGillis W. R. (1999). A cubic relationship
between air-sea CO2 exchange and
wind speed. Geophys. Res. Lett..

[11] Wurl O., Holmes M. (2008). The gelatinous nature of the sea-surface microlayer. Mar. Chem..

[12] Cunliffe M., Engel A., Frka S., Gašparović B., Guitart C., Murrell J. C., Salter M., Stolle C., Upstill-Goddard R., Wurl O. (2013). Sea surface microlayers: A unified
physicochemical and biological perspective of the air–ocean
interface. Prog. Oceanogr..

[13] Sabbaghzadeh B., Upstill-Goddard R., Beale R., Pereira R., Nightingale P. D. (2017). The Atlantic
Ocean surface microlayer from 50 N to 50 S is ubiquitously enriched
in surfactants at wind speeds up to 13 m s– 1. Geophys. Res. Lett..

[14] Wurl O., Wurl E., Miller L., Johnson K., Vagle S. (2011). Formation
and global distribution of sea-surface microlayers. Biogeosciences.

[15] Cunliffe M., Salter M., Mann P. J., Whiteley A. S., Upstill-Goddard R. C., Murrell J. C. (2009). Dissolved organic
carbon and bacterial populations
in the gelatinous surface microlayer of a Norwegian fjord mesocosm. FEMS Microbiol. Lett..

[16] Häder D. P., Kumar H., Smith R., Worrest R. (2007). Effects of solar UV
radiation on aquatic ecosystems and interactions with climate change. Photochem. Photobiol. Sci..

[17] Pereira R., Ashton I., Sabbaghzadeh B., Shutler J. D., Upstill-Goddard R. C. (2018). Reduced
air–sea CO 2 exchange in the Atlantic Ocean due to biological
surfactants. Nat. Geosci..

[18] Holding T., Ashton I. G., Shutler J. D., Land P. E., Nightingale P. D., Rees A. P., Brown I., Piolle J.-F., Kock A., Bange H. W. (2019). The
FluxEngine air–sea gas flux toolbox:
simplified interface and extensions for in situ analyses and multiple
sparingly soluble gases. Ocean Sci..

[19] Pereira R., Schneider-Zapp K., Upstill-Goddard R. (2016). Surfactant control of gas transfer
velocity along an offshore coastal transect: results from a laboratory
gas exchange tank. Biogeosciences.

[20] Frew N. M., Goldman J. C., Dennett M. R., Johnson A. S. (1990). Impact of phytoplankton-generated
surfactants on air-sea gas exchange. J. Geophys.
Res.:Oceans.

[21] Burdette T. C., Bramblett R. L., Deegan A. M., Coffey N. R., Wozniak A. S., Frossard A. A. (2022). Organic Signatures of Surfactants and Organic Molecules
in Surface Microlayer and Subsurface Water of Delaware Bay. ACS Earth Space Chem..

[22] Wurl O., Miller L., Röttgers R., Vagle S. (2009). The distribution and
fate of surface-active substances in the sea-surface microlayer and
water column. Mar. Chem..

[23] Frew N. M., Nelson R. K., McGillis W. R., Edson J. B., Bock E. J., Hara T. (2002). Spatial variations
in surface microlayer surfactants and their role
in modulating air-sea exchange. Geophys. Monogr.
Ser..

[24] Cincinelli A., Stortini A. M., Perugini M., Checchini L., Lepri L. (2001). Organic pollutants
in sea-surface microlayer and aerosol in the coastal
environment of Leghorn—(Tyrrhenian Sea). Mar. Chem..

[25] Schneider-Zapp K., Salter M. E., Upstill-Goddard R. (2014). An automated
gas exchange tank for
determining gas transfer velocities in natural seawater samples. Ocean Sci..

[26] Edson, J. B. ; Fairall, C. ; Bariteau, L. ; Zappa, C. J. ; Cifuentes-Lorenzen, A. ; McGillis, W. R. ; Pezoa, S. ; Hare, J. ; Helmig, D. Direct covariance measurement of CO2 gas transfer velocity during the 2008 Southern Ocean Gas Exchange Experiment: Wind speed dependency J. Geophys. Res.:Oceans 2011; Vol. 116 C4 10.1029/2011JC007022.

[27] Ho, D. T. ; Wanninkhof, R. ; Schlosser, P. ; Ullman, D. S. ; Hebert, D. ; Sullivan, K. F. Toward a universal relationship between wind speed and gas exchange: Gas transfer velocities measured with 3He/SF6 during the Southern Ocean Gas Exchange Experiment J. Geophys. Res.:Oceans 2011; Vol. 116 C4 10.1029/2010JC006854.

[28] Ribas-Ribas M., Kilcher L. F., Wurl O. (2018). Sniffle: a step forward
to measure
in situ CO2 fluxes with the floating chamber technique. Elem.:Sci. Anthropocene.

[29] Upstill-Goddard, R. C. ; Frost, T. ; Henry, G. R. ; Franklin, M. ; Murrell, J. C. ; Owens, N. J. Bacterioneuston control of air-water methane exchange determined with a laboratory gas exchange tank Global Biogeochem. Cycles 2003; Vol. 17 4 10.1029/2003GB002043.

[30] Zappa, C. J. ; McGillis, W. R. ; Raymond, P. A. ; Edson, J. B. ; Hintsa, E. J. ; Zemmelink, H. J. ; Dacey, J. W. ; Ho, D. T. Environmental turbulent mixing controls on air-water gas exchange in marine and aquatic systems Geophys. Res. Lett. 2007; Vol. 34 10 10.1029/2006GL028790.

[31] Pajala G., Rudberg D., Gålfalk M., Melack J. M., Macintyre S., Karlsson J., Sawakuchi H. O., Schenk J., Sieczko A., Sundgren I. (2023). Higher
Apparent Gas Transfer Velocities for
CO2 Compared to CH4 in Small Lakes. Environ.
Sci. Technol..

[32] Upstill-Goddard R., Rees A., Owens N. (1996). Simultaneous high-precision measurements
of methane and nitrous oxide in water and seawater by single phase
equilibration gas chromatography. Deep Sea Res.,
Part I.

[33] Hunt C. W., Snyder L., Salisbury J. E., Vandemark D., McDowell W. H. (2017). SIPCO2: A simple, inexpensive surface
water pCO2 sensor. Limnol. Oceanogr.:Methods.

[34] Martin C.
R., Zeng N., Karion A., Dickerson R. R., Ren X., Turpie B. N., Weber K. J. (2017). Evaluation and environmental correction
of ambient CO 2 measurements from a low-cost NDIR sensor. Atmos. Meas. Tech..

[35] Müller M., Graf P., Meyer J., Pentina A., Brunner D., Perez-Cruz F., Hüglin C., Emmenegger L. (2020). Integration
and calibration of non-dispersive infrared (NDIR) CO 2 low-cost sensors
and their operation in a sensor network covering Switzerland. Atmos. Meas. Tech..

[36] Rickard P. C., Uher G., Upstill-Goddard R. C. (2022). Photo-Reactivity
of Surfactants in
the Sea-Surface Microlayer and Subsurface Water of the Tyne Estuary,
UK. Geophys. Res. Lett..

[37] Wanninkhof R., Asher W. E., Ho D. T., Sweeney C., McGillis W. R. (2009). Advances
in quantifying air-sea gas exchange and environmental forcing. Annu. Rev. Mar. Sci..

[38] Weiss R. F. (1974). Carbon
dioxide in water and seawater: the solubility of a non-ideal gas. Mar. Chem..

