# Supplementary material for: GETCO2: A Compact and Portable System for Automated Gas Transfer Velocity Measurements of Natural Waters
Source: ACS Earth Space Chem. 2026 Feb 24;10(3):711–9. doi: 10.1021/acsearthspacechem.5c00329 (PMC13007037; doi:10.1021/acsearthspacechem.5c00329)
Supplement: Supplementary file 1 [file sp5c00329_si_001.pdf]

Supporting Information for Publication

GETCO2: A Compact and Portable System for Automated Gas Transfer Velocity Measurements of Natural Waters

Authors:

Sevda Norouzi[1*] (SN), S.Norouzi_Alibabalou@hw.ac.uk

Ryan Pereira[1*] (RP), R.Pereira@hw.ac.uk

[*]Corresponding authors

[1]The Lyell Centre, School of Energy, Geoscience, Infrastructure and Society, Heriot-Watt University, Research Avenue South, Edinburgh, EH14 4AS, United Kingdom

Supporting Information for Publication

Appendix A: Gaussian error propagation for $\frac{L}{\beta}$

$$\sigma_{C_w}^2 = \left(\frac{C_e - C_{aa}}{V_{ws}}\right)^2 \sigma_{V_h}^2 + \left(\frac{V_h(C_e - C_{aa})}{{V_{ws}}^2}\right)^2 \sigma_{V_{ws}}^2 + C_e\sigma_\alpha^2 + \alpha^2\sigma_{C_e}^2 + \left(\frac{V_h}{V_{ws}}\right)^2 \sigma_{C_{aa}}^2 \tag{1}$$

$$\sigma_D^2 = \sigma_{C_w}^2 + \alpha^2\sigma_{C_a}^2 + C_a\sigma_\alpha^2 \tag{2}$$

$$\sigma_L^2 = \frac{1}{D^2}\sigma_D^2 + \frac{1}{{D_0}^2}\sigma_{D_0}^2 \tag{3}$$

$$\sigma_\beta^2 = (\frac{1}{{h_w}^2})^2 \sigma_{h_w}^2 + (\frac{\alpha}{{h_a}^2})^2 \sigma_{h_a}^2 + (\frac{1}{{h_a}^2})^2 \sigma_\alpha^2 \tag{4}$$

$$\sigma_{L/\beta}^2 = \frac{1}{\beta^2}^2 \sigma_L^2 + (\frac{L}{\beta^2})^2 \sigma_\beta^2 \tag{5}$$

Appendix B: Gaussian error propagation for $\frac{L_n}{B_n}$

$$\sigma^2_{h_{w\,s_n}} = (\frac{t_{p\,n}}{A})^2 \sigma^2_{Q_p} + (\frac{t_{p\,n} * Q_p}{A^2})^2 \sigma^2_A \tag{6}$$

$$\sigma^2_{\beta_n} = (\frac{1}{(h_{w\,0} - \sum_{i=1}^{n} h_{w\,s_n})^2})^2 \sigma^2_{h_{w\,0}} + \sum_{j=1}^{n} (\frac{1}{(h_{w\,0} - \sum_{i=1}^{j} h_{w\,s_i})^2})^2 \sigma^2_{h_{w\,s_j}} \tag{7}$$

$$\sigma^2_{B_n} = \sum_{j=1}^{n} \beta_j (\frac{t_j - t_{j-1}}{t_n - t_0})^2 \sigma^2_{\beta_j} \tag{8}$$

$$\sigma^2_{\frac{L_n}{B_n}} = (\frac{1}{B_n})^2 \sigma^2_{L_n} + (\frac{L_n}{{B_n}^2})^2 \sigma^2_{B_n} \tag{9}$$

Supporting Information for Publication

Appendix C: Datasets used for the generation of Figures 2 and 3

Table S1: Datasets used for the generation of Figures 2a-c

a)	10 ppmv standard gas
Time (s)	Sensor reading (a.u.)
32.02	431
34.03	386
36.04	342
42.07	245
44.08	221
46.09	197
48.10	174
54.13	124
56.14	111
58.15	101
60.16	95
66.19	77
68.20	72
70.21	67
72.22	60
78.25	51
80.26	49
82.27	47
84.28	44
90.31	38
92.32	35
94.33	33
96.34	32
102.37	28
104.38	27
106.39	25
108.40	23
114.43	22
116.44	22
118.45	22
120.46	21
126.49	20
128.50	19
130.51	19
132.52	19
138.55	18
140.56	18
142.57	18
144.58	17
150.61	17
152.62	17

154.63	18
156.64	18
162.67	17
164.68	17
166.69	18
168.70	18
174.73	18
176.74	18
178.75	18
180.76	18
186.79	19
188.80	19
190.81	19
196.84	29
198.85	36
200.86	45
202.87	59
208.90	86
210.91	93
212.92	94
214.93	95
220.96	98
222.97	100
224.98	101
226.99	101
233.02	102
235.03	101
237.04	101
239.05	101
245.08	100
247.09	99
249.10	98
251.11	95
257.14	91
259.15	89
261.16	88
263.17	87
269.20	82
271.21	80
273.22	78
275.23	77
281.26	73
283.27	72
285.28	71
287.29	70
293.32	67

295.33	66
297.34	66
299.35	65
305.38	63
307.39	63
309.40	62
311.41	62
317.44	60
319.45	60
321.46	60
323.47	60
329.50	59
331.51	58
333.52	57
335.53	58
341.56	56
343.57	56
345.58	56
347.59	56
353.62	56
355.63	55
357.64	56
359.65	56
365.68	56
392.69	56
394.70	55
396.71	55
402.74	64
404.75	74
406.76	86
408.77	102
414.80	151
416.81	156
418.82	163
420.83	166
426.86	184
428.87	190
430.88	195
432.89	199
438.92	204
440.93	207
442.94	208
444.95	209
450.98	214
452.99	216
455.00	217

457.01	218
463.04	221
465.05	223
467.06	223
469.07	223
475.10	225
477.11	226
479.12	226
481.13	225
487.16	226
489.17	226
491.18	227
493.19	228
499.22	228
501.23	228
503.24	228
505.25	229
511.28	229
513.29	229
515.30	230
517.31	229
523.34	230
525.35	230
527.36	230
529.37	230
535.40	230
537.41	230
539.42	230
541.43	230
547.46	230
549.47	230
551.48	230
553.49	230
559.52	231
561.53	231
563.54	231
565.55	231
571.58	231
603.59	220
605.60	210
611.63	144
613.64	124
615.65	111
617.66	100
623.69	78
625.70	75

627.71	66
629.72	61
635.75	48
637.76	45
639.77	42
641.78	39
647.81	36
649.82	35
651.83	34
653.84	33
659.87	28
661.88	27
663.89	27
665.90	26
671.93	22
673.94	22
675.95	21
677.96	21
683.99	21
686.00	21
688.01	21
690.02	20
696.05	19
698.06	19
700.07	19
702.08	19
708.11	18
710.12	18
712.13	18
714.14	17
720.17	17
722.18	17
724.19	17
730.22	19
732.23	21
734.24	26
736.25	31
742.28	59
744.29	65
746.30	69
748.31	73
754.34	85
756.35	86
758.36	87
760.37	87
766.40	91

768.41	91
770.42	91
772.43	90
778.46	90
780.47	90
782.48	90
784.49	90
790.52	87
792.53	85
794.54	85
796.55	85
802.58	81
804.59	80
806.60	78
808.61	77
814.64	73
816.65	72
818.66	70
820.67	70
826.70	68
828.71	68
830.72	67
832.73	66
838.76	64
840.77	64
842.78	63
844.79	63
850.82	62
852.83	61
854.84	61
856.85	60
862.88	59
864.89	57
866.90	57
868.91	58
874.94	56
876.95	56
878.96	56
880.97	56
887.00	55
889.01	54
891.02	54
893.03	54
899.06	55
901.07	54
903.08	54

930.09	51
936.12	54
938.13	58
940.14	65
942.15	75
948.18	124
950.19	135
952.20	140
954.21	147
960.24	167
962.25	172
964.26	177
966.27	181
972.30	191
974.31	196
976.32	196
978.33	198
984.36	203
986.37	205
988.38	206
990.39	207
996.42	212
998.43	212
1000.44	213
1002.45	213
1008.48	216
1010.49	216
1012.50	218
1014.51	219
1020.54	221
1022.55	222
1024.56	222
1026.57	222
1032.60	223
1034.61	223
1036.62	223
1038.63	223
1044.66	224
1046.67	224
1048.68	224
1050.69	224
1056.72	224
1058.73	225
1060.74	225
1062.75	225
1068.78	225

1070.79	226
1072.80	225
1074.81	226
1080.84	226
1082.85	226
1084.86	226
1086.87	226
1092.90	226
1094.91	226
1096.92	226
1098.93	226
1104.96	226
1106.97	226
1108.98	227
1140.99	220
1147.02	170
1149.03	148
1151.04	125
1153.05	115
1159.08	82
1161.09	79
1163.10	75
1165.11	69
1171.14	55
1173.15	50
1175.16	46
1177.17	45
1183.20	39
1185.21	36
1187.22	35
1189.23	34
1195.26	30
1197.27	29
1199.28	29
1201.29	27
1207.32	26
1209.33	25
1211.34	24
1213.35	24
1219.38	23
1221.39	21
1223.40	21
1225.41	21
1231.44	20
1233.45	20
1235.46	20

1237.47	20
1243.50	20
1245.51	19
1247.52	19
1249.53	19
1255.56	18
1257.57	18
1259.58	18
1261.59	18
1267.62	20
1269.63	24
1271.64	29
1273.65	37
1279.68	62
1281.69	69
1283.70	73
1285.71	77
1291.74	86
1293.75	89
1295.76	89
1297.77	89
1303.80	92
1305.81	93
1307.82	93
1309.83	93
1315.86	93
1317.87	93
1319.88	92
1321.89	91
1327.92	88
1329.93	87
1331.94	86
1333.95	85
1339.98	80
1341.99	79
1344.00	78
1346.01	76
1350.45	78
1352.46	76
1354.47	74
1356.48	74
1362.51	71
1364.52	70
1366.53	69
1368.54	69
1374.57	68

1376.58	68
1378.59	67
1380.6	67
1386.64	66
1388.65	65
1390.66	65
1392.67	64
1398.7	64
1400.71	63
1402.72	63
1404.73	62
1410.76	61
1412.77	61
1414.78	61
1416.79	61
1422.82	60
1424.83	60
1426.84	60
1428.85	60
1434.88	60
1436.89	60
1438.9	60
1440.91	60
1446.94	60
1448.95	60
1450.96	60
1452.97	60
1459	59
1461.01	59
1463.02	59
1465.03	59
1471.06	59
1473.07	59
1500.08	59
1502.09	59
1508.12	64
1510.13	68
1512.14	78
1514.15	88
1520.18	145
1522.19	155
1524.2	160
1526.21	166
1532.24	182
1534.25	187
1536.26	188

1538.27	191
1544.3	198
1546.31	202
1548.32	204
1550.33	207
1556.36	213
1558.37	214
1560.38	215
1562.39	216
1568.42	220
1570.43	222
1572.44	222
1574.45	223
1580.48	224
1582.49	225
1584.5	226
1586.51	226
1592.54	227
1594.55	228
1596.56	228
1598.57	227
1604.6	228
1606.61	227
1608.62	227
1610.63	227
1616.66	228
1618.67	228
1620.68	228
1622.69	228
1628.72	229
1630.73	229
1632.74	229
1634.75	229
1640.78	229
1642.79	229
1644.8	229
1646.81	230
1652.84	230
1654.85	230
1656.86	230
1658.87	230
1664.9	230
1666.91	230
1668.92	230
1670.93	230
1676.96	231

Supporting Information for Publication

b)	1000 ppmv standard gas
Time (s)	Sensor reading (a.u.)
30.01	567
32.02	565
34.03	565
36.04	566
38.05	573
40.06	585
46.09	698
48.10	740
50.11	774
52.12	796
54.13	827
56.14	849
58.15	876
60.16	887
62.17	899
64.18	910
66.19	918
68.20	927
70.21	934
72.22	942
78.25	973
80.26	980
82.27	983
84.28	984
86.29	990
88.30	992
90.31	997
92.32	1001
94.33	1004
96.34	1005
98.35	1008
100.36	1009
102.37	1009
104.38	1010
106.39	1010
112.42	1017
114.43	1017
116.44	1018
118.45	1017
120.46	1019
122.47	1019
124.48	1020
126.49	1021
128.50	1021

130.51	1021
132.52	1022
134.53	1021
140.56	1023
142.57	1023
144.58	1022
146.59	1022
148.60	1022
150.61	1021
152.62	1021
154.63	1021
156.64	1022
158.65	1021
160.66	1021
162.67	1021
164.68	1022
166.69	1021
168.70	1021
170.71	1021
172.72	1021
174.73	1020
176.74	1018
178.75	1010
180.76	1001
182.77	993
188.80	969
190.81	964
192.82	958
194.83	953
196.84	948
198.85	946
200.86	944
202.87	944
204.88	941
206.89	942
208.90	945
210.91	946
212.92	947
218.95	951
220.96	952
222.97	954
224.98	959
226.99	964
229.00	967
231.01	969
233.02	972

235.03	977
237.04	979
239.05	980
241.06	984
243.07	985
253.12	993
255.13	997
257.14	999
259.15	1003
261.16	1006
263.17	1007
265.18	1009
267.19	1009
269.20	1011
271.21	1011
273.22	1014
275.23	1016
277.24	1018
279.25	1018
281.26	1016
287.29	1021
289.30	1024
291.31	1027
293.32	1029
295.33	1030
297.34	1029
299.35	1031
301.36	1031
303.37	1030
305.38	1030
307.39	1030
309.40	1030
311.41	1030
313.42	1030
315.43	1030
317.44	1030
319.45	1030
321.46	1029
323.47	1031
325.48	1031
327.49	1031
329.50	1031
331.51	1031
333.52	1032
335.53	1031
337.54	1033

339.55	1034
345.58	1033
347.59	1032
349.60	1032
376.61	1030
378.62	1022
380.63	1015
382.64	1003
384.65	990
386.66	978
388.67	957
394.70	908
396.71	900
398.72	891
400.73	882
402.74	876
404.75	873
406.76	868
408.77	867
410.78	863
412.79	857
414.80	855
416.81	854
418.82	853
420.83	850
426.86	845
428.87	841
430.88	840
432.89	838
434.90	837
436.91	835
438.92	836
440.93	834
442.94	833
444.95	833
446.96	832
448.97	831
459.02	827
461.03	826
463.04	824
465.05	823
467.06	825
469.07	823
471.08	821
473.09	820
475.10	818

477.11	819
479.12	819
481.13	819
487.16	818
489.17	818
491.18	819
493.19	818
495.20	817
497.21	817
499.22	817
501.23	819
503.24	818
505.25	819
507.26	820
509.27	819
511.28	819
517.31	822
519.32	821
551.33	849
553.34	858
555.35	864
557.36	881
559.37	898
561.38	911
563.39	919
565.40	928
567.41	940
569.42	948
571.43	954
573.44	960
579.47	971
585.50	985
587.51	988
589.52	989
591.53	995
593.54	998
595.55	1001
597.56	1005
599.57	1007
601.58	1007
603.59	1007
605.60	1008
607.61	1010
609.62	1011
611.63	1011
613.64	1010

615.65	1009
617.66	1010
619.67	1010
621.68	1011
623.69	1011
625.70	1011
627.71	1010
629.72	1010
631.73	1010
633.74	1010
635.75	1011
637.76	1011
639.77	1012
641.78	1012
643.79	1013
645.80	1015
647.81	1013
649.82	1013
651.83	1011
653.84	1010
655.85	1005
657.86	1000
659.87	998
661.88	997
663.89	995
665.90	990
667.91	988
669.92	985
671.93	984
673.94	982
675.95	981
677.96	977
679.97	977
681.98	975
683.99	975
686.00	974
688.01	975
690.02	977
692.03	977
694.04	977
696.05	979
702.08	982
704.09	983
706.10	984
708.11	984
710.12	987

712.13	987
714.14	989
716.15	992
718.16	993
720.17	995
722.18	997
724.19	1001
726.20	1002
728.21	1004
730.22	1005
732.23	1008
734.24	1010
736.25	1011
738.26	1010
740.27	1011
742.28	1011
744.29	1011
746.30	1011
748.31	1011
750.32	1012
752.33	1013
754.34	1013
756.35	1014
758.36	1015
760.37	1016
762.38	1017
764.39	1016
766.40	1017
768.41	1017
770.42	1018
772.43	1017
774.44	1017
776.45	1016
782.48	1016
784.49	1016
786.50	1016
788.51	1016
790.52	1016
792.53	1016
794.54	1016
796.55	1016
798.56	1018
800.57	1018
802.58	1019
804.59	1018
806.60	1018

833.61	1019
835.62	1015
837.63	1010
839.64	1002
841.65	987
843.66	972
845.67	954
847.68	933
849.69	919
851.70	908
853.71	892
855.72	887
857.73	883
859.74	878
861.75	874
867.78	860
869.79	855
871.80	850
873.81	846
875.82	845
877.83	844
879.84	842
881.85	839
883.86	836
885.87	833
887.88	832
889.89	832
891.90	830
893.91	828
895.92	826
897.93	827
899.94	826
901.95	824
903.96	824
905.97	823
907.98	823
909.99	822
912.00	821
914.01	821
916.02	822
918.03	821
920.04	820
922.05	820
924.06	820
926.07	819
928.08	818

930.09	818
932.10	818
934.11	818
936.12	818
938.13	817
940.14	816
942.15	816
948.18	818
950.19	818
952.20	818
954.21	818
956.22	817
958.23	818
960.24	818
980.24	849
982.24	858
984.24	864
986.24	881
988.24	898
990.24	911
992.24	919
994.24	928
996.24	940
998.24	948
1000.24	954
1002.24	960
1008.24	971
1014.24	985
1016.24	988
1018.24	989
1020.24	995
1022.24	998
1024.24	1001
1026.24	1005
1028.24	1007
1030.24	1007
1032.24	1007
1034.24	1008
1036.24	1010
1038.24	1011
1040.24	1011
1042.24	1010
1044.24	1009
1046.24	1010
1048.24	1010
1050.24	1011

1052.24	1011
1054.24	1011
1056.24	1010
1058.24	1010
1060.24	1010
1062.24	1010
1064.24	1011
1066.24	1011
1072.24	1012
1074.24	1012
1076.24	1013
1078.24	1015
1080.24	1013
1082.24	1013
1084.24	1011
1086.24	1010
1088.24	1005
1090.24	1000
1092.24	998
1094.24	997
1096.24	998
1098.24	990
1100.24	998
1102.24	998
1104.24	994
1106.24	992
1108.24	981
1110.24	997
1112.24	977
1114.24	975
1116.24	975
1118.24	974
1120.24	975
1122.24	977
1124.24	977
1126.24	977
1128.24	979
1134.24	982
1136.24	983
1138.24	984
1140.24	984
1142.24	987
1144.24	987
1146.24	989
1148.24	992
1150.24	993

1152.24	995
1154.24	997
1156.24	1001
1158.24	1002
1160.24	1004
1162.24	1005
1164.24	1008
1166.24	1010
1168.24	1011
1170.24	1010
1172.24	1011
1174.24	1011
1176.24	1011
1178.24	1011
1180.24	1011
1182.24	1012
1184.24	1013
1186.24	1013
1188.24	1014
1190.24	1015
1192.24	1016
1194.24	1017
1196.24	1016
1198.24	1017
1200.24	1017
1202.24	1018
1204.24	1017
1206.24	1017
1208.24	1016
1224.24	1016
1226.24	1016
1228.24	1016
1230.24	1018
1232.24	1018
1234.24	1019
1236.24	1018
1238.24	1018
1240.24	1019
1244.24	1019
1246.24	1018
1248.24	1018
1250.24	1019
1252.24	1020
1254.24	1018
1274.24	1019
1276.24	1015

1278.24	1010
1280.24	1002
1282.24	987
1284.24	972
1286.24	954
1288.24	933
1290.24	919
1292.24	908
1294.24	892
1296.24	887
1298.24	883
1300.24	878
1302.24	874
1308.24	860
1310.24	855
1312.24	850
1314.24	846
1316.24	845
1318.24	844
1320.24	842
1322.24	839
1324.24	836
1326.24	833
1328.24	832
1330.24	832
1332.24	830
1334.24	828
1336.24	826
1338.24	827
1340.24	826
1342.24	824
1344.24	824
1346.24	823
1348.24	823
1350.24	822
1352.24	821
1354.24	821
1356.24	822
1358.24	821
1360.24	820
1362.24	820
1364.24	820
1366.24	819
1368.24	818
1370.24	818
1372.24	818

1374.24	818
1376.24	818
1378.24	817
1380.24	816
1382.24	816
1388.24	818
1390.24	818
1392.24	818
1394.24	818
1396.24	817
1398.24	818
1400.24	818
c)	3000 ppmv standard gas
Time (s)	Sensor reading (a.u.)
40.46	1615
42.53	1772
44.59	1915
46.66	2028
48.72	2116
50.82	2183
52.89	2263
54.97	2324
57.03	2368
59.10	2402
61.16	2418
63.22	2447
65.36	2460
67.42	2466
69.51	2469
71.57	2471
73.63	2471
75.70	2476
77.77	2479
79.90	2486
81.96	2485
84.04	2489
86.11	2490
88.17	2490
90.23	2490
92.30	2490
94.44	2490
100.65	2495
102.72	2498
104.78	2497
106.84	2501
108.97	2500

111.04	2501
113.12	2505
115.19	2503
117.25	2504
119.33	2502
121.39	2500
123.51	2500
125.57	2500
127.66	2502
129.72	2503
131.78	2502
133.85	2500
135.91	2501
138.05	2502
140.11	2502
142.19	2506
144.26	2507
146.32	2506
148.38	2503
150.45	2502
152.59	2502
154.65	2503
156.73	2502
158.81	2502
160.87	2502
162.94	2502
165.00	2504
167.12	2504
173.38	2503
175.44	2509
177.51	2528
179.57	2546
181.71	2560
183.77	2564
185.83	2564
187.90	2553
189.96	2541
192.03	2525
194.09	2507
196.20	2499
198.26	2481
200.36	2467
202.42	2458
204.48	2448
206.55	2444
208.61	2439

210.73	2437
212.80	2433
214.88	2436
216.95	2436
219.01	2436
221.07	2436
223.14	2437
225.27	2440
227.34	2440
229.42	2446
231.48	2451
233.55	2455
235.62	2458
237.68	2461
239.81	2465
241.87	2463
243.96	2464
246.02	2465
248.08	2471
250.15	2473
252.21	2476
254.35	2479
256.41	2482
258.49	2482
260.56	2485
262.62	2486
264.68	2489
266.75	2494
268.88	2496
270.95	2495
273.03	2494
275.09	2494
277.16	2494
279.22	2496
281.31	2495
283.42	2496
285.49	2496
287.57	2496
289.64	2497
291.70	2499
293.77	2500
295.83	2501
297.96	2502
300.02	2504
302.11	2503
304.17	2505

306.23	2505
308.30	2506
310.36	2507
312.50	2508
314.56	2507
316.64	2509
318.71	2510
320.77	2510
322.85	2512
324.92	2511
327.03	2509
329.10	2511
331.18	2513
333.24	2513
335.31	2512
337.37	2512
339.44	2512
341.57	2511
343.63	2511
345.72	2511
347.78	2510
349.85	2510
351.91	2513
353.98	2514
356.11	2515
358.17	2516
360.26	2517
362.33	2517
364.40	2518
366.46	2517
368.54	2518
370.65	2518
372.71	2518
374.79	2520
376.86	2519
378.92	2518
411.06	2508
413.13	2398
415.26	2271
421.48	2134
423.54	2144
425.61	2148
427.67	2155
429.80	2155
436.02	2165
438.08	2166

440.14	2170
442.22	2173
444.34	2179
446.40	2178
448.48	2177
450.55	2177
452.61	2177
454.67	2175
456.74	2174
458.87	2172
460.94	2174
463.02	2174
465.08	2178
467.15	2176
469.21	2176
471.28	2175
473.41	2173
475.48	2173
477.56	2173
479.62	2172
481.69	2172
483.76	2166
485.83	2163
487.95	2161
490.01	2162
492.10	2158
494.16	2160
496.22	2158
498.29	2158
500.35	2157
502.49	2156
508.71	2155
510.77	2156
512.85	2156
514.92	2157
517.02	2157
519.09	2158
521.17	2160
523.24	2160
525.30	2160
527.38	2160
529.44	2162
531.56	2160
533.63	2160
535.71	2164
537.77	2162

539.84	2162
541.90	2163
543.96	2161
546.10	2160
592.45	2380
594.51	2400
596.58	2422
598.64	2445
600.74	2472
602.80	2483
604.89	2488
606.95	2492
609.01	2493
611.09	2495
613.16	2498
615.28	2498
617.34	2503
619.42	2503
621.49	2508
623.55	2510
625.61	2512
627.68	2514
629.82	2515
631.88	2517
633.98	2516
636.04	2517
638.10	2516
640.17	2517
642.23	2517
644.35	2518
646.42	2518
648.50	2517
650.57	2518
652.63	2519
654.71	2518
656.78	2518
658.89	2520
660.96	2521
663.04	2520
665.11	2520
667.17	2521
669.23	2522
671.30	2522
673.43	2522
679.68	2515
681.75	2513

683.81	2515
685.87	2520
687.97	2521
690.03	2524
692.11	2520
694.18	2522
696.25	2523
698.32	2517
700.38	2518
702.50	2517
704.57	2515
706.65	2515
708.71	2510
710.78	2509
712.84	2512
714.92	2511
717.04	2508
719.10	2507
721.19	2507
723.25	2507
725.31	2507
727.38	2507
729.44	2507
731.58	2507
733.64	2508
735.72	2507
737.79	2507
739.87	2507
741.93	2507
743.99	2507
746.12	2506
748.18	2506
750.26	2506
752.33	2507
754.40	2507
756.47	2508
758.53	2509
760.65	2508
796.96	2137
799.03	2068
801.09	2045
803.15	2040
805.27	2053
807.33	2058
809.42	2074
811.48	2078

813.56	2083
815.63	2091
817.69	2094
819.81	2097
821.87	2102
823.97	2109
826.03	2112
828.09	2119
830.16	2123
832.22	2129
834.34	2134
836.41	2137
838.49	2140
840.55	2137
842.62	2138
844.68	2140
846.74	2140
848.88	2139
850.95	2134
853.03	2131
855.11	2131
857.17	2132
859.23	2133
861.30	2130
863.42	2130
869.64	2131
871.70	2130
873.77	2128
875.83	2126
877.96	2122
880.02	2120
882.10	2119
884.17	2122
886.23	2120
888.29	2119
890.36	2117
892.49	2118
894.56	2115
896.64	2114
898.72	2113
900.78	2112
902.84	2112
904.92	2113
907.03	2113
909.09	2113
911.18	2114

913.24	2115
915.30	2115
917.37	2115
919.43	2115
921.57	2115
923.63	2117
965.86	2278
967.93	2325
970.00	2357
972.06	2388
974.13	2414
976.26	2440
978.35	2453
980.42	2469
982.49	2479
984.57	2490
986.63	2494
988.74	2500
990.80	2500
992.89	2503
994.95	2503
997.02	2505
999.08	2506
1001.14	2507
1003.28	2511
1005.34	2510
1007.43	2509
1009.50	2509
1011.56	2512
1013.62	2513
1015.69	2515
1017.82	2516
1019.88	2517
1021.96	2516
1024.03	2515
1026.12	2516
1028.18	2516
1030.24	2516
1032.35	2516
1034.42	2515
1036.50	2515
1038.56	2516
1040.63	2517
1042.69	2518
1044.75	2518
1046.89	2518

1053.15	2517
1055.21	2516
1057.27	2518
1059.34	2519
1061.48	2520
1063.54	2521
1065.62	2522
1067.69	2521
1069.76	2522
1071.82	2522
1073.96	2519
1076.02	2515
1078.11	2512
1080.17	2509
1082.23	2507
1084.30	2507
1086.36	2507
1088.50	2507
1090.56	2505
1092.64	2506
1094.71	2507
1096.78	2507
1098.86	2507
1100.93	2507
1103.04	2508
1105.10	2507
1107.19	2507
1109.26	2507
1111.34	2510
1113.40	2511
1115.46	2511
1117.57	2510
1119.64	2511
1121.72	2511
1123.78	2510
1125.85	2509
1127.91	2509
1129.97	2510
1132.11	2511
1134.17	2511
1166.34	2505
1168.41	2334
1170.47	2142
1172.54	2068
1174.60	2058
1176.72	2062

1178.79	2070
1180.87	2076
1182.94	2082
1185.03	2087
1187.09	2093
1189.16	2096
1191.26	2106
1193.33	2116
1195.41	2124
1197.47	2134
1199.54	2136
1201.60	2140
1203.66	2144
1205.80	2144
1207.86	2144
1209.95	2145
1212.01	2144
1214.07	2144
1216.16	2145
1218.22	2145
1220.34	2142
1222.40	2142
1224.48	2142
1226.55	2141
1228.62	2142
1230.69	2141
1232.75	2139
1234.87	2137
1236.94	2135
1239.02	2133
1241.08	2131
1243.15	2127
1245.22	2126
1247.28	2124
1249.41	2121
1251.47	2120
1253.56	2120
1255.62	2115
1257.70	2114
1259.76	2116
1261.83	2113
1263.95	2109
1266.01	2106
1268.09	2105
1270.17	2106
1272.24	2106

1274.30	2106
1276.36	2108
1278.49	2109
1280.55	2119
1282.63	2105
1284.70	2108
1286.76	2109
1288.82	2108
1290.89	2108
1292.95	2112
1295.02	2114
1297.08	2114
1299.14	2115
1301.21	2118
1303.27	2114
1305.34	2119
1307.40	2109
1309.46	2115

Table S2: Datasets used for the generation of Figures 2d-f

Standard gas CO2 concentration (ppmv)	Sensor reading (a.u.)		
	Air	Ambient air	Bubbling
10	18	56	230
1000	1014	1023	820
3000	2511	2512	2128

Table S3: Datasets used for the generation of Figures 3

time (hr)	Water CO2 concentration (ppmv)	Air CO2 concentration (ppmv)	Ln/Bn (cm)	Relative total mass
0.00	4245	770	0.00	1.00
0.18	3730	1191	-1.50	0.98
0.36	3458	1502	-2.75	0.98
0.54	3203	1759	-4.20	0.98
0.72	3106	1932	-5.20	1.00
0.91	3034	2043	-6.02	1.01
1.08	2962	2121	-6.81	1.01

Table S4: GETCO2 specifications of data in Figures 3

Specifications	
Salinity (ppt)	0.00
Water tmperature (C)	15.90
Initial water height (m)	0.23
Initial air height (m)	0.22
Water pump rate (ml/s)	3.88
Air pump rate (ml/s)	6.00
Water pump work duration per cycle (s)	40.00
Air pump work duration per cycle (s)	10.00
